# Enhancing Bioenergy Production from the Raw and Defatted Microalgal Biomass Using Wastewater as the Cultivation Medium

**DOI:** 10.3390/bioengineering9110637

**Published:** 2022-11-02

**Authors:** Gang Li, Yuhang Hao, Tenglun Yang, Wenbo Xiao, Minmin Pan, Shuhao Huo, Tao Lyu

**Affiliations:** 1 School of Artificial Intelligence, Beijing Technology and Business University, Beijing 100048, China; 2 Department for Solar Materials, Helmholtz Centre for Environmental Research GmbH-UFZ, Permoserstraße 15, 04318 Leipzig, Germany; 3 School of Food and Biological Engineering, Jiangsu University, Zhenjiang 212013, China; 4 School of Water, Energy and Environment, Cranfield University, College Road, Cranfield MK43 0AL, UK

**Keywords:** biodiesel, waste recovery, renewable energy, microalgal technology, life cycle assessment

## Abstract

Improving the efficiency of using energy and decreasing impacts on the environment will be an inevitable choice for future development. Based on this direction, three kinds of medium (modified anaerobic digestion wastewater, anaerobic digestion wastewater and a standard growth medium BG11) were used to culture microalgae towards achieving high-quality biodiesel products. The results showed that microalgae culturing with anaerobic digestate wastewater could increase lipid content (21.8%); however, the modified anaerobic digestion wastewater can boost the microalgal biomass production to 0.78 ± 0.01 g/L when compared with (0.35–0.54 g/L) the other two groups. Besides the first step lipid extraction, the elemental composition, thermogravimetric and pyrolysis products of the defatted microalgal residues were also analysed to delve into the utilisation potential of microalgae biomass. Defatted microalgae from modified wastewater by pyrolysis at 650 °C resulted in an increase in the total content of valuable products (39.47%) with no significant difference in the content of toxic compounds compared to other groups. Moreover, the results of the life cycle assessment showed that the environmental impact (388.9 mPET_2000_) was lower than that of raw wastewater (418.1 mPET_2000_) and standard medium (497.3 mPET_2000_)-cultivated groups. Consequently, the method of culturing microalgae in modified wastewater and pyrolyzing algal residues has a potential to increase renewable energy production and reduce environmental impact.

## 1. Introduction

In total, 84% of the world’s energy demand is still met by non-renewable energy sources, and the ever-growing energy consumption is depleting the total fossil energy storage [1]. In the meantime, the consequences of the large utilization of fossil fuels have led to serious environmental issues, such as toxic compound discharge, global warming, the extinction of species, desertification etc. [2]. Renewable energy could contribute significantly to adjusting the global energy structure, thus, has been regarded as the inevitable choice for sustainable development. Renewable energy includes biomass energy, wind energy, solar energy, water energy, geothermal energy, etc., and has the characteristics of wide resource distribution, great utilization potential and small environmental impacts. Among these, biomass energy is a significant source of renewable carbon that can be transformed into endless conventional solid, liquid, and gaseous fuels [3].

Traditional biomass energy sources, e.g., crops, are always criticized due to the concern of land use competition for food production and slower rates of plant growth [4]. Microalgae, as a major class of biomass energy, are outstanding attributed to their various advantages, e.g., rapid growth, high lipid content, carbon sequestration ability, and low demands of environmental conditions (barren land, saline- and waste-water) [5]. The esters and glycerol rich in microalgae are good raw materials for the preparation of liquid fuels. The calorific value of bio-oil prepared by microalgae pyrolysis is relatively high. Moreover, the nutrients from wastewater can be effectively used by microalgae for its biomass growth. Consequently, the concept of utilizing wastewater for the cultivation of microalgae has drawn much attention in terms of the simultaneous treatment of wastewater and producing renewable energy.

Effluent from anaerobic digestion reactors has been seen as an ideal medium for microalgal growth regarding the reduced level of organics and variety of residual nutrients (e.g., N, P) in the liquid [6]. Although the challenge of lacking key elements in the wastewater can bring a negative impact on the growth of microalgae [7], the approach of artificial wastewater adjustment, e.g., addition of a certain amount of missing elements, has been deployed and proven to be an efficient way to address this problem to cultivate a good amount of algal biomass [8]. When considering the entire life cycle, barriers such as algal residues after lipid extraction have hitherto hindered the application of microalgal biomass energy. Algal residues account for about 70% of the dry weight of microalgae biomass and still contain large amounts of carbohydrates and proteins [9]. In most studies, the subsequent utilization of algae residues was not mentioned after culturing microalgae and extracting lipids. The energy held by photosynthesis in the biomass may be recovered quickly and cleanly using the pyrolysis method, which outperforms biological methods in terms of effectiveness, cost, and energy balance [10]. Therefore, quantifying the potential of biofuel generation from defatted algal biomass would be important for renewable energy production.

Besides the biofuel generation from microalgal biomass, the environmental impact of the pyrolysis process needs to be assessed to identify any trade-offs [11]. Some drawbacks have been reported; for example, the relative contents of nitrogen compounds in the residues of defatted microalgae could increase during the pyrolysis [12]. Furthermore, the addition of a catalyst would increase the contents of CO in pyrolysis products of *Haematococcus pluvialis* residues [13]. Moreover, the pyrolysis of *Isochrysis* after oil extraction required 2591 kJ/kg of energy [14]. The whole process of bioenergy production from microalgae residues can be fully understood by using life cycle assessment (LCA) [15,16]. In different culture conditions, such as using different wastewaters or modified wastewater, the compositions of microalgae and subsequent microalgae residues would be impacted [17]. Consequently, the properties of the compositions of microalgae have a direct effect on pyrolysis products [18]. The assessment of the pyrolysis of microalgae residues gained by different culture methods has not hitherto been sufficiently investigated.

In this study, the algae strain *Desmodesmus* sp. was chosen as the model microalgal species and cultured in modified anaerobic digestion wastewater (MAW), original anaerobic digestion wastewater (AW), and BG11 media, respectively. The lipid was firstly extracted from the microalgae cultivated in three culture medias, then, the algae residues were pyrolyzed at a temperature range of 350 °C to 750 °C. The lipid contents and compositions of microalgae were compared, and the differences in thermal decomposition behaviour of three kinds of microalgae residues were evaluated. Through the evaluation of the pyrolysis products, the optimum pyrolysis temperature was determined, and the life cycle assessment was carried out. This study provides a new insight into the thorough utilization of microalgae to produce high-quality biodiesel through the improvement of the microalgae culture medium and the reuse of the defatted microalgal biomass. A follow-up LCA analysis provides an environmentally sustainable view of the proposed strategy. The results have great significance to the improvement of the microalgae culture medium, the optimization of the technological process of biofuel production, and the strategy of environmental control in the pyrolysis process.

## 2. Materials and Methods

### 2.1. Algal Strain and Culture Conditions

*Desmodesmus sp*. EJ 8-10 (hereinafter referred to as EJ 8-10) is an algal strain that was identified in a river in Beijing, China. With an initial inoculum ratio of 10% (*v*/*v*), EJ 8-10 was pre-cultivated in flasks (250 mL). The following steps were the precise cultivation conditions: BG11 medium (autoclaved) (Appendix A, Table A1); lighting intensity: 120 ± 2 mmol/m^2^/s; temperature: 27 ± 1 °C; lighting period: 14 h:10 h (light:dark); pH: 7.5 [19]. Anaerobic digestion wastewater (AW) was gathered from a pig farm in the Shunyi District of Beijing. The supernatant was collected for microalgae cultivation after centrifugation (10,000 rpm, 15 min). The composition is shown in Table A2 (Appendix B). High concentrations of NH_4_^+^–N could reduce microalgal vitality; the collected supernatant was diluted to 10% with deionized water [20]. To address the nutritional deficit in wastewater, the modified anaerobic digestion wastewater (MAW) medium was created by additionally adding ammonium ferric citrate (C_6_H_8_FeNO_7_), dipotassium hydrogenphosphate (K_2_HPO_4_), and magnesium sulfate heptahydrate (MgSO_4_·7H_2_O) [21]. The standard medium of BG11 was used as a control group, and 0.1 OD_680_ of initial microalgal biomass was inoculated into three mediums (AW, MAW, and BG11) and cultured for 14 days under identical circumstances as previously indicated. The experiments were run three times for each group.

### 2.2. Lipid Extraction and Fatty Acid Analysis

After culture, microalgae were collected by centrifuging at 10,000 rpm for 10 min. The total lipid content was then determined by using an improved approach based on Abou-Shanab et al. [22]. The mixed solvent (volume ratio of chloroform, methanol, and water was 1:2:0.8) was added to ground algae powder (mass ratio of algae powder to quartz sand was 1:3) and then oscillated for 5 min [23]. After standing for 15 min, the mixture was centrifuged (6000 rpm, 2 min) and the upper extract was collected. After the above operation was repeated for precipitation two times, all extracts were merged. Extracts were mixed thoroughly with chloroform, methanol, and water until the final volume ratio was 1:1:0.9. Chloroform solution was gathered following delamination of the combination. Solvent chloroform was evaporated in a rotary evaporator (vacuum, 60 °C), and the obtained lipid was weighed. We collected algae residues (hereinafter referred to as AR) and used them for subsequent pyrolysis to prepare bio-oil. The experiments were carried out three times for each group. The lipid contents were calculated by the following formula:

(1) C=W1/Wb×100%

where the lipid mass (mg) is *W*1, the algae mass (mg) is *Wb*, and the lipid content (%) is *C*.

Preparative FAMEs and gas chromatography–mass spectrometry (GC–MS) analyses were used to assess the fatty acid composition. The preparation process of FAMEs refers to a method of Wang et al. [24]. We added 10 mL of the mixture (methanol, concentrated sulfuric acid, and chloroform had a volume ratio of 4.25:0.75:5) to a screw-top glass bottle (25 mL) containing 0.1 g of the sample [25]. In a 90 °C water bath (Cole-Parmer, Vernon Hills, IL, USA), transesterification was performed for 90 min. A meticulous collection of the FAME-containing chloroform layer was made for GC–MS analysis. A flame ionization detector and an RTX-Wax capillary column (30 m 0.32 mm 0.25 mm; Restek Corp., Bellefonte, PA, USA) were placed in the GC (QP2010; Shimadzu Corp., Kyoto, Japan). The oven’s temperature was initially set at 100 °C (held for three minutes), then was increased to 200 °C at 4 °C/min, and increased to 250 °C (held for five minutes) at 3 °C/min. The carrier gas (helium) flow rate was regulated at 30 mL/min, and the injector temperature was fixed at 230 °C. The NIST Mass Spectral Database was used to identify the FAME compounds, and the peak regions of the compounds were compared to those of the external standard (C18:2) (Sigma Aldrich, Saint Louis, MN, USA) to determine their amounts [19]. The experiments were conducted three times for each group.

### 2.3. Elemental Analysis, Thermogravimetric Analysis (TGA), and Pyrolysis of Algal Residues

Each lipid-extracted AR sample’s primary elements composition (C, H, N, and S) was determined using an elemental analyzer (EA; Flash EA-1112, Thermo Scientific, Waltham, MA, USA). The experiments were performed three times for each group. Using Equations (2)–(4), the higher heating values (HHV) of AR samples were determined [26,27]:

(2) $$HHV(OLS)=1.87C^{2}-144C-2082H+63.8C\times H+129N+20147$$

(3) $$HHV(PLS)=5.22C^{2}-319C-1674H+38.6C\times H+133N+21028$$

(4) $$\begin{array}{l} HHV=\left[HHV(OLS)+HHV(PLS)\right]/2\\ =(3.55C^{2}-232C-2230H+51.2C\times H+131N+20600)\times 10^{-3} \end{array}$$

where *C*, *H*, and *N* stand for the sample’s respective carbon, hydrogen, and nitrogen contents (%), respectively.

In the TGA procedure, nitrogen (99.999% purity, 100 mL/min) was used as a shielding gas while 2–4 mg samples were pyrolyzed from 50 °C to 800 °C at 20 °C/min. The samples were pyrolyzed and examined using pyrolysis–gas chromatography–mass spectrometry (Py–GC–MS), which consists of a rapid pyrolyzer (Frontier Labs 3030i, Koriyama, Fukushima, Japan) coupled with a GC–MS (Agilent 7890A/5975C, Santa Clara, CA, USA) in order to describe thermal decomposition behaviour and pyrolysis products of AR. 

The pyrolysis products at various pyrolysis temperatures (350 °C, 450 °C, 550 °C, 650 °C, and 750 °C) were examined to find the best pyrolysis conditions for AR. The GC–MS was operating under the same circumstances as before. Pyrolysis products were located by scanning the NIST11 database (National Institute of Standards and Technology, Gaithersburg, MD, USA) for the resulting mass spectra [28].

### 2.4. Life Cycle Assessment (LCA)

To achieve a more comprehensive overview of energy consumption and environmental impacts caused by the pyrolysis process (under corresponding optimal temperature) of ARs obtained under different culture conditions, an LCA investigation was conducted [11]. The study’s chosen objectives and field of inquiry were compliant with the international standards for life cycle assessments, i.e., ISO 14040 [29]. 

#### 2.4.1. LCA Goals and System Boundaries

**Figure 1:**
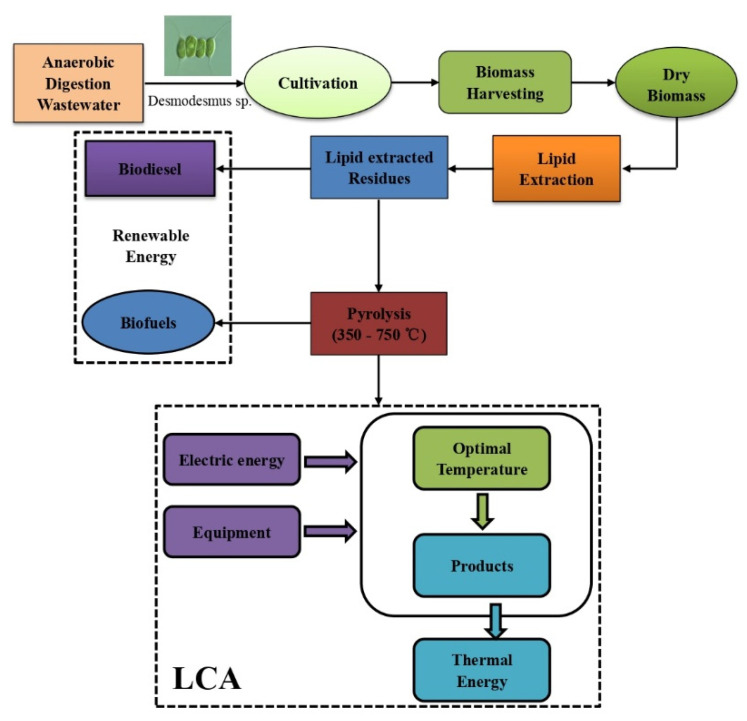
Process route and system boundaries of the LCA model for pyrolysis products of microalgal residues.

Utilizing LCA serves the objective of assessing the environmental impact of AR gathered in various media on the manufacture of the best pyrolysis products. Figure 1 depicts the boundary of the system, and the energy consumption during system operation is regarded as the input. The depreciation of pyrolysis equipment, the energy consumption of adding additional nutrients to the modified medium, and the impact of microalgae growth on the environment were excluded [30]. It is worth noting that the pollutants in the process were not treated as extras.

#### 2.4.2. Selected Parameters to Describe the Environmental Impacts

The evaluation details four aspects of environmental impact, including photochemical ozone synthesis (kg VOC, CO, CH_4_-eq), acidification (kg SO_2_, NO_X_-eq), eutrophication (kg PO_4_, NO_X_-eq), and global warming (kg CO_2_, CH_4_, NO_X_, CO-eq) [31]. The energy consumption and environmental impact was determined using 1 kg biomass dry weight EJ 8–10 residue for assessment.

#### 2.4.3. LCA Stages

In order to examine the pyrolysis stage of AR, this study’s assessment ignored the energy transfer in the pyrolyzer and instead focused on the energy consumption of the pyrolysis furnace and online analysis [30]. The average energy consumption is 2.2 kWh for each real-time analysis. Based on the pyrolysis product results, the energy required to raise the temperature from ambient (25.6 °C) to each group’s optimum conditions was estimated, and air pollutant emissions were determined using data from a prior study [11].

#### 2.4.4. LCA Model

The formula below was used to determine environmental impact.:

(5) $$EI=\sum_{}^{}\left[Q_{i}\times F_{i}\right]$$

where *EI* stands for the environmental impact, *Q_i_* for the ith emission’s quantity, and *F_i_* for the influence of the *i*th emission on the environment as a whole [11].

Equation (6) was used to further standardize the EI for the comparative assessment of various sorts of impacts [30]:

(6) $$S_{EI}=EI\times R^{-1}$$

where *R* is the accepted benchmark and *S_EI_* stands for the standardized environmental effect.

The weighting factor was determined by the method of target distance as the following equation (Equation (7)):

(7) $$W=E\times {EN}^{-1}$$

the unit (mPET2000) of standardized environmental potential impact is represented by the standard person equivalent, and *W* is the weighting factor for each particular parameter. *E* is the overall regional environmental impact potential in 1990, whereas *EN* is the regional environmental impact potential in 2000 [31].

### 2.5. Plotting and Statistical Analysis

Origin 9.8 (OriginLab, Northampton, Massachusetts, USA) was used for plotting, and data analysis was done by using SPSS 19.0 (IBM Corporation, Armonk, NY, USA). Prior to statistical analysis, the data were examined for normality and homogeneity of variance. Nonparametric test methods were employed for the analysis if the variables were not normally distributed. If variables were normally distributed, the F-test (ANOVA) was used to assess the significant difference in data. The significance level was 0.05. Data are presented as mean ± standard deviation.

## 3. Results and Discussion

### 3.1. Microalgal Growth, Lipid Accumulation and Fatty Acid Composition

**Figure 2:**
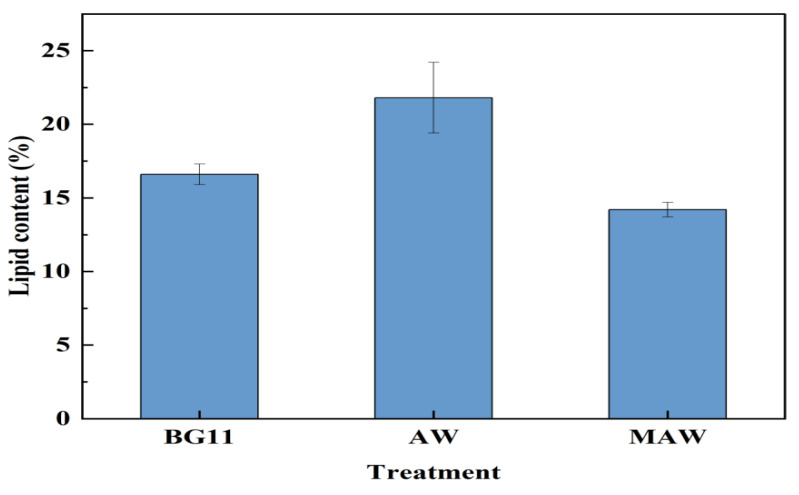
Lipid contents of three kinds of microalgae (obtained from BG11, AW and MAW medium, respectively).

Overall, culturing microalgae with AW can obtain the highest lipid accumulation (21.8%, Figure 2). Furthermore, the microalgae cultured in the modified anaerobic digestion wastewater (MAW) had the highest biomass production (0.78 ± 0.01 g/L) compared with those (0.35–0.54 g/L) in the other two groups. The outcomes demonstrated that anaerobic digestion of wastewater could increase lipid accumulation when used to cultivate microalgae. Tan et al. [32] explained that the anaerobic wastewater had balanced nutrients with many trace elements that were not present in the medium, but the low content of trace elements such as P, Fe and Mg in AW could not guarantee the rapid growth of microalgae. Those nutrients are all necessary for photosynthesis at the growth stages of microalgae, therefore, the biomass production of microalgae with the addition of P and Mg was the highest in MAW [32]. However, the lipid content (14.2%) of microalgae decreased after culturing in MAW. The main elements added to the modified medium were Mg and P, and P could promote lipid accumulation [33]. However, Mg was related to photosynthesis, and microalgae might first choose to accumulate carbohydrates instead of lipids in the case of sufficient carbon sources [34]. Therefore, it was possible that lipid content was lower in modified wastewater. In this experiment, the lipid productivity of microalgae in MAW was higher than that found in the study by Chinnasamy et al. [35], which may be due to the nutrient shortage in the medium during the microalgae growth.

**Figure 3:**
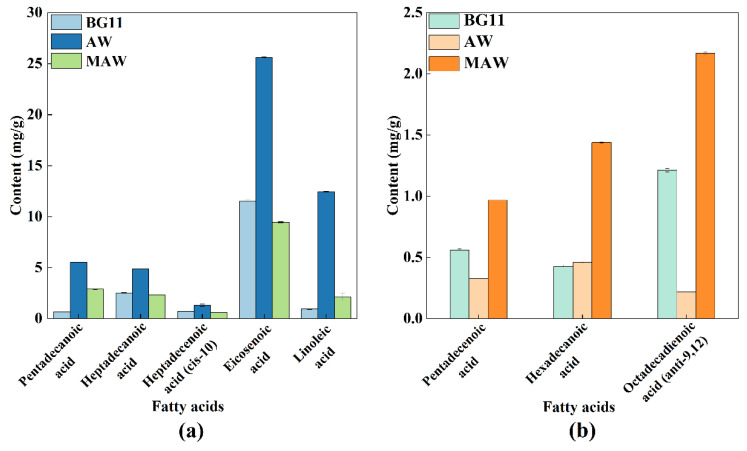
Contents of fatty acids in microalgae harvested by the three culture methods; (**a**) Pentadecanoic acid, Heptadecanoic acid, Heptadecenoic acid (cis-10), Eicosenoic acid and Linoleic acid, and (**b**) Pentadecenoic acid, Hexadecanoic acid and Octadecadienoic acid (anti-9,12).

The first step of lipid extraction was conducted to evaluate the content of different compositions. The AW group had the largest amounts of saturated fatty acids (SFAs), polyunsaturated fatty acids (PUFAs), and monounsaturated fatty acids (MUFAs) of the three groups (Appendix C, Table A3). These three fatty acids were frequently used in skin care products, and when cultured with AW, their production was boosted (Figure 3a). When compared with *Desmodesmus sp.* cultured with 10% original wastewater (48.37 mg/g) in the study of Li et al. [19], the total fatty acids content was lower than that of microalgae harvested from the AW medium (74.74 mg/g); higher P content in AW medium may contribute to the higher fatty acids content in the microalgae. Microalgae cultured in wastewater had advantages in their contents of pentadecanoic acid, heptadecanoic acid, heptadecenoic acid (cis-10), eicosenoic acid and linoleic acid (Figure 3a), among which pentadecanoic acid, margaric acid, paullinic acid and linoleic acid are widely used in medical, pharmaceutical, and nutritional fields [36]. Heptadecenoicacid (cis-10) could balance the low temperature resistance and combustion performance of biodiesel, and play an important role in the production of biodiesel. In addition, the contents of pentadecenoic acid, hexadecanoic acid and octadecadienoic acid (anti-9,12) were significantly increased (*p* < 0.05) after culturing microalgae with modified wastewater (Figure 3b). These fatty acids are important industrial raw materials for the preparation of medicines, emulsifiers, and detergents, respectively. Hexadecanoic acid and octadecadienoic acid (anti-9,12) were two of the most suitable biofuel sources extracted from microalgae, and ARs obtained from modified anaerobic digestion wastewater were advantageous for these molecules, which coincide with the research of Wang et al. [24]. The lipid contents of microalgae from MAW (129.20 mg/g) were higher than those cultured with original piggery effluent (48.37 mg/g); the MAW (4.91 mg/L) contained higher P contents compared with the original piggery effluent (3.10 mg/L), which could theoretically contribute to a higher accumulation of lipid content in the microalgae [19]. Similar to the research of Moradianetal et al. [3], microalgal biomass has received much attention and esteem because of their powerful capabilities in various aspects of life and industry.

### 3.2. Properties and Thermogravimetric Analysis of Microalgal Residues

Though with economic advantages, AW could significantly (*p* < 0.05) reduce the content of C, H, and N, leading to a decreased HHV when compared with the BG11 group. However, MAW could address this issue by increasing the C and H content to 47.24 ± 0.01% and 7.49 ± 0.28%, respectively. Notably, the contents of C and H were the key parameters determining the HHV of material, and the AR of MAW had a higher HHV, indicating a higher energy density, which would promote further pyrolysis products. The samples of Huang et al. [12] showed no significant difference in HHV before and after lipid extraction, indicating a similar energy potential of the material and residues of microalgae. It was clear from the results that further studies to convert microalgal residues into energy-related products are feasible.

**Figure 4:**
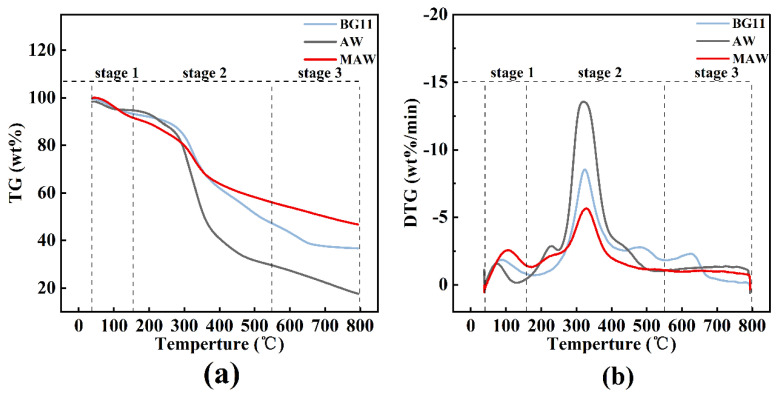
(**a**) TG and, (**b**) DTG analysis of pyrolysis process.

TGA data, meanwhile (Figure 4a), added to the argument. In comparison to those harvested in BG11 and AW, microalgal residues harvested in MAW medium showed a slower rate of weight loss throughout the pyrolysis stage. Due to the continuing decompositions and carbonizations of AR, TG decreased slowly at the stage above 550 °C [37]. Microalgal residues obtained from the MAW had the highest contents of C (Table 1), which improved the thermal resistance of the AR and resulted in the highest amounts of thermal residues (46.75 wt%) [38]. Notably, the contents of N and S were also elevated in the group of MAW, indicating a potential higher production of harmful compounds, e.g., NO_x_, SO_2_, and HCN, during pyrolysis or subsequent combustion and upgrading processes. Therefore, a pyrolysis compound analysis for both valuable and toxic compounds was conducted.

### 3.3. Valuable and Toxic Pyrolysis Products of Algal Residues

Ingredients of the medium and pyrolysis temperatures could affect pyrolysis products, and forasmuch, the present study compared the pyrolysis products of microalgal residues from different microalgae media (BG11, AW and MAW) and different pyrolysis temperatures (over the range 350–750 °C). Consistent with other research results [39], aromatic hydrocarbons (benzenes, indenes, and their derivatives), aliphatic hydrocarbons (alkanes and olefins), phenols, fatty acids, nitrogen-containing compounds (amides, nitriles, and pyridines), polycyclic aromatic hydrocarbons (PAHs), and other trace components made up the majority of the pyrolysis products of ARs (ketones, alcohols, aldehydes, and furans).

#### 3.3.1. Valuable Compounds

**Figure 5:**
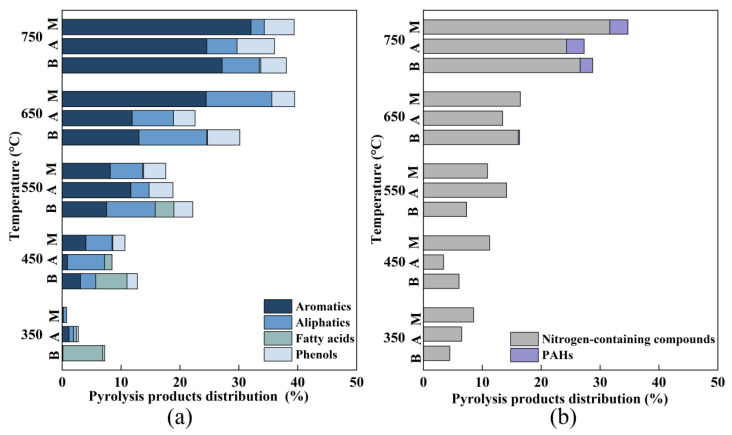
In the pyrolysis of microalgal leftovers grown on BG11, AW and MAW media; (**a**) useful chemicals, and (**b**) hazardous compounds were produced (A: AW; B: BG11; M: MAW). The numerical results are shown in Table A4 (Appendix D).

Among all pyrolysis products, aliphatic hydrocarbons are some of the products with economic value, which have great significance in the production of bio-oils. The percentage of valuable compounds in the pyrolysis products of microalgal residues from the MAW medium increased as the pyrolysis temperature rose from 350 to 750 °C and showed a single peak at 650 °C (39.47%, Figure 5a, Table A4 in Appendix D); compared with results of the BG11 (38.08%, 750 °C) and AW (36.05%, 750 °C) groups, MAW groups significantly reduced the pyrolysis temperature of optimal content and increased the relative content of valuable compounds in the pyrolysis products. The content of valuable compounds obtained by pyrolysis in microalgae residues from the MAW medium (39.47%) was higher than that from original anaerobically digested effluent medium (19.83%). The results agreed with the previous study that the content of valuable compounds from pyrolysis production in MAW medium increase by adding chemical components (ammonium ferric citrate, dipotassium hydrogenphosphate, and magnesium sulfate heptahydrate) [12]. Moreover, aromatic hydrocarbons in pyrolysis products are also high-value compounds, which can be used as transportation fuel additives in industry, and can also elevate octane numbers, thereby enhancing combustion efficiency [40]. The results showed that contents of aromatic hydrocarbons in the pyrolysis products of three groups of AR showed similar trends; aromatic hydrocarbons were the most abundant components in all pyrolysis products, and achieved the maximum value at 750 °C with the increase of pyrolysis temperature. The contents of aromatic hydrocarbons in the pyrolysis products of ARs in both BG11 (27.16%) and AW (24.54%) media were all lower than those in the MAW (32.07%) groups by comparison.

#### 3.3.2. Toxic Compounds

Due to insufficient breakdown during pyrolysis, microalgae leftovers may potentially produce hazardous chemicals such as nitrogen-containing compounds and polycyclic aromatic hydrocarbons (PAHs), in addition to beneficial molecules, affecting the quality of bio-oil products and affecting environmental pollution with excessive emission of nitrogen oxides [41]. In the current investigation, there was a strong association (r = 0.93, 0.89, 0.87; *p* < 0.01) between pyrolysis temperatures and the relative concentrations of nitrogen-containing chemicals; the contents of nitrogen-containing compounds and PAHs produced by pyrolysis of microalgae residues in MAW groups (16.48%, 0%; 650 °C) were lower than those in the BG11 groups (26.60%, 2.15%; 750 °C) and AW groups (24.33%, 2.97%; 750 °C) at the temperature at which the maximum quantity of valuable pyrolysis products emerged. Except for nitrogen-containing compounds and PAHs, sulphides were also toxic products to consider. The ARs of MAW groups had the highest sulphur contents (0.95 ± 0.01%, Table 1), indicating that there might be a high potential for the production of sulphur dioxide, SO_2_, during the pyrolysis process, and further improvement was needed [42].

In conclusion, when compared to BG11 and AW media, ARs from the MAW medium promoted higher levels of valuable compounds (such as aromatic hydrocarbons and aliphatic hydrocarbons), while the levels of toxic substrates (such as nitrogen-containing compounds and PAHs) were not significantly different (*p* > 0.05). It is important to note that choosing the ideal pyrolysis temperature involves more than just balancing the needs of the goal product with the trade-offs between harmful substances, valuable products, and energy consumption.

### 3.4. Life Cycle Assessment of Microalgal Residues Pyrolysis

**Figure 6:**
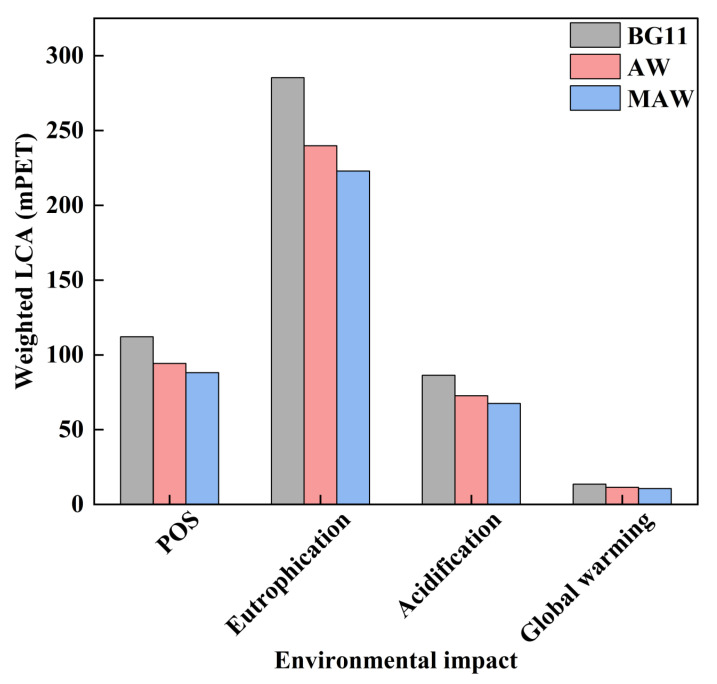
Weighted life cycle assessment of three kinds of medium. POS represents the impact of photochemical ozone synthesis.

LCA was applied to the process of pyrolysis of microalgal residues in three media at the optimum pyrolysis temperature (650 °C), and the pyrolysis of ARs in the BG11 medium required the most energy (7463.5 MJ/kg); directly using anaerobic digestion wastewater (AW) reduced the required energy by 15.93%, and in the modified anaerobic digestion wastewater (MAW) group, it was further reduced by 7.09%. This was mostly related to an increase in biomass output in wastewater that had been modified and included more plentiful and balanced nutrients for microalgal growth [31]. The pyrolysis products from MAW groups generated overall lower environmental impacts (total, 388.9 mPET_2000_, Figure 6) for all four parameters selected, and led to a lower total environmental impact than the pyrolysis processes of AR obtained from AW (total, 418.1 mPET_2000_) and BG11 (497.3 mPET_2000_) media. The relative distribution of each environmental category during the pyrolysis operations did not differ significantly (*p* > 0.05) across the various groups (Figure 6). Eutrophication, which was primarily brought on by NO_x_ emissions, was induced by all groups’ combined largest contributions (285.3, 239.8, and 222.8 mPET_2000_ by BG11, AW, and MAW, respectively) [41]. While the microalgae were given the ability to photosynthesise, the effects of pyrolysis product creation on global warming (13.6, 11.4, and 10.6 mPET_2000_ by BG11, AW, and MAW, respectively) were minimal. In summary, compared with the other two cultivation methods, modified anaerobic digestion wastewater could not change the proportion of a single environmental impact, but could significantly reduce total environmental impacts.

### 3.5. Future Perspectives

In the process of microalgae growth, C source and N sources are the main nutrient sources for microalgae. The effluent of anaerobic digestion is a cheap and directly applicable nutrient source, and the cost of microalgae culture will be greatly reduced. In a real biogas project, while producing clean energy, the anaerobic digestion broth could be used to culture microalgae [43]. After extracting oil, the pyrolytic algae residues could also convert wastes into industrial available energy. This could solve problems of the high transportation cost of anaerobic digestion broth, as well as increased pollution to the surrounding environment in biogas engineering. Industries could avoid the loss and waste of beneficial elements, and produce new bioenergy at the same time, which could not only meet the goal of sustainable development, but also reduce carbon emissions. In the conversion process of biodiesel, biomass needs to be separated or purified. The transesterification reaction requires the installation of methanol recovery equipment, and due to the formation of soap, the treatment process is complex, and the purification of the product is difficult. In addition, with the increase of contents of S and N in ARs, harmful substances produced during pyrolysis will increase, such as sulphides and nitrogen-containing compounds [44]. The technological process should be further optimized to avoid the increase of harmful substances and increase the content of bio-oil at the same time.

## 4. Conclusions

The modified anaerobic digestion wastewater (MAW), through the extra additions of C_6_H_8_FeNO_7_, K_2_HPO_4_, and MgSO_4_·7H_2_O, could significantly promote lipid quality in microalgal biomass, towards enhancing the quality of biofuels. The modified medium also improved the composition and properties of microalgal residues after the first step of lipid extraction, resulting in higher thermal resistance (thermal residue was 46.75 wt%) when compared to that from anaerobic digestion wastewater (AW) (17.61 wt%) and the standard BG11 (36.72 wt%) cultivation medium. MAW groups significantly reduced the pyrolysis temperature (650 °C) of optimal content and increased the relative content of valuable pyrolysis products, of which aliphatic hydrocarbons were 1.9 and 2 times as abundant as other groups, respectively, along with decreasing the contents of toxic compounds (nitrogen-containing compounds and PAHs) in products. Moreover, compared with the other two cultivation methods, MAW could not change the proportion of single environmental impacts, but could significantly reduce total environmental impacts when compared with the BG11 (reduced by 21.79%) and AW (reduced by 6.97%) groups, as indicated by the LCA.

## Figures and Tables

**Table 1:** Elemental analyses and the higher heating values (HHV) of microalgae biomass cultivated in BG11, AW and MAW media.

	C (%)	H (%)	N (%)	S (%)	HHV (MJ/kg)
BG11	35.01 ± 0.06	5.97 ± 0.01	8.04 ± 0.03	0.60 ± 0.01	15.27 ± 0.01
AW	21.87 ± 0.04	4.93 ± 0.05	5.81 ± 0.03	0.68 ± 0.11	12.51 ± 0.05
MAW	47.24 ± 0.01	7.49 ± 0.28	11.25 ± 0.16	0.95 ± 0.01	20.45 ± 0.07

## Data Availability

Not applicable.

## Appendix A

**Table A1:** The components of BG11 medium.

No.	Chemicals	Concentration (g/L)
1	NaNO_3_	1.5
2	K_2_HPO_4_	3 × 10^−2^
3	MgSO_4_·7H_2_O	7.5 × 10^−2^
4	CaCl_2_·2H_2_O	36 × 10^−2^
5	Iron Citrate	6 × 10^−3^
6	Ammonium Citrate	6 × 10^−3^
7	EDTA	1 × 10^−3^
8	Na_2_CO_3_	6 × 10^−3^
9	H_3_BO_3_	2.86 × 10^−3^
	MnCl_2_·4H_2_O	1.81 × 10^−3^
	ZnSO_4_·7H_2_O	2.22 × 10^−4^
	NaMoO_4_·5H_2_O	3.9 × 10^−4^
	CuSO_4_·5H_2_O	7.9 × 10^−5^
	Co(NO_2_)_2_·6H_2_O	4.94 × 10^−4^

## Appendix B

**Table A2:** The components of 10% (v/v) AW.

Components	Concentration (mg/L)
NH_4_^+^-N	42.8
PO_4_^3−^	2.09
TN	50.92
TP	2.31

## Appendix C

**Table A3:** Compositions of fatty acids, including saturated fatty acids (SFA), monounsaturated fatty acids (MUFA) and polyunsaturated fatty acids (PUFA), of microalgae cultivated in BG11, AW and MAW medium, respectively.

Fatty Acids Sort	Fatty Acids	Fatty Acids Form	BG11		AW		MAW	
			mg/g	%	mg/g	%	mg/g	%
SFA	Tannic acid	C10:0	0.576 ± 0.007	0.35	0.599 ± 0.003	0.27	0.514 ± 0.001	0.36
	Hendecanoic acid	C11:0	0.417 ± 0.003	0.25	0.272 ± 0.001	0.12	0.211 ± 0.001	0.15
	Dodecanoic acid	C12:0	3.809 ± 0.037	2.29	2.618 ± 0.004	1.20	1.518 ± 0.006	1.07
	Tridecanoic acid	C13:0	1.017 ± 0.025	0.61	0.177 ± 0.002	0.08	0.199 ± 0.001	0.14
	Myristic acid	C14:0	0.391 ± 0.006	0.24	0.249 ± 0.003	0.11	0.171 ± 0.003	0.12
	Pentadecanoic acid	C15:0	0.655 ± 0.006	0.39	5.514 ± 0.008	2.53	2.892 ± 0.015	2.04
	Hexadecanoic acid	C16:0	0.936 ± 0.010	0.56	0.976 ± 0.002	0.45	0.462 ± 0.003	0.33
	Heptadecanoic acid	C17:0	2.537 ± 0.033	1.53	4.897 ± 0.006	2.25	2.350 ± 0.011	1.66
	Octadecanic acid	C18:0	0.363 ± 0.007	0.22	0.939 ± 0.003	0.43	0.609 ± 0.002	0.43
	Henicosanoic acid	C21:0	1.879 ± 0.052	1.13	0.262 ± 0.004	0.12	0.237 ± 0.001	0.17
MUFA	Tetradecenoic acid (cis-9)	C14:1	0.364 ± 0.004	0.22	0.437 ± 0.005	0.20	0.431 ± 0.003	0.30
	Pentadecenoic acid	C15:1	0.561 ± 0.009	0.34	0.326 ± 0.002	0.15	0.968 ± 0.005	0.68
	Hexadecanoic acid	C16:1	0.428 ± 0.005	0.26	0.460 ± 0.003	0.21	1.440 ± 0.004	1.01
	Heptadecenoic acid (cis-10)	C17:1	0.716 ± 0.010	0.43	1.310 ± 0.110	0.60	0.618 ± 0.003	0.44
	Octadecenoic acid (cis-9)	C18:1n9c	1.518 ± 0.024	0.91	1.799 ± 0.006	0.83	1.302 ± 0.006	0.92
	Eicosenoic acid	C20:1	11.501 ± 0.124	6.93	25.597 ± 0.081	11.74	9.459 ± 0.052	6.66
PUFA	Octadecadienoic acid (cis-9,12)	C18:2n6t	14.219 ± 0.326	8.57	13.026 ± 0.066	5.98	11.550 ± 0.043	8.13
	Octadecadienoic acid (anti-9,12)	C18:2n6c	1.213 ± 0.015	0.73	0.219 ± 0.001	0.10	2.168 ± 0.011	1.53
	Octadecatrienoic acid (cis-9,12,15)	C18:3n6	1.100 ± 0.020	0.66	0.239 ± 0.001	0.11	0.157 ± 0.001	0.11
	Linoleic acid	C18:3n3	0.944 ± 0.031	0.57	12.457 ± 0.041	5.71	2.126 ± 0.357	1.50
	Eicosadienoic acid (cis-11,14)	C20:2	0.211 ± 0.003	0.13	0.689 ± 0.002	0.32	0.210 ± 0.001	0.15
	Eicosatrienoic acid (cis-8,11,14)	C20:3n6	0.266 ± 0.006	0.16	0.262 ± 0.002	0.12	0.242 ± 0.002	0.17
	Arachidonic acid (cis-5,8,11,14)	C20:4n6	0.290 ± 0.001	0.17	0.502 ± 0.003	0.23	0.126 ± 0.001	0.09
	Docosadienoic acid (cis-13,16)	C20:2	0.876 ± 0.022	0.53	0.918 ± 0.036	0.42	0.442 ± 0.010	0.31

## Appendix D

**Table A4:** The product contents of microalgae residues obtained from three kinds of medium (BG11, AW and MAW) at different pyrolysis temperatures.

Medium	Pyrolysis Products	Temperature				
	(%)	350 °C	450 °C	550 °C	650 °C	750 °C
BG11	Aliphatics	0.13	2.61	8.25	11.51	6.29
	Aromatics	0.00	3.11	7.54	13.03	27.16
	Fatty acids	6.76	5.30	3.18	0.15	0.27
	Phenols	0.34	1.76	3.20	5.47	4.36
	Nitrogen-containing compounds	4.50	6.06	7.35	16.10	26.60
	PAHs	0.00	0.00	0.00	0.23	2.15
AW	Aliphatics	0.76	6.32	3.10	7.03	5.16
	Aromatics	1.17	0.90	11.66	11.89	24.54
	Fatty acids	0.48	1.26	0.00	0.00	0.00
	Phenols	0.34	0.00	4.05	3.66	6.35
	Nitrogen-containing compounds	6.52	3.43	14.14	13.46	24.33
	PAHs	0.00	0.00	0.00	0.00	2.97
MAW	Aliphatics	0.55	4.45	5.50	11.13	2.25
	Aromatics	0.19	4.03	8.16	24.46	32.07
	Fatty acids	0.00	0.13	0.18	0.00	0.00
	Phenols	0.00	2.06	3.75	3.88	5.08
	Nitrogen-containing compounds	8.52	11.26	10.88	16.48	31.64
	PAHs	0.00	0.00	0.00	0.00	3.10
